# Linguistic Skills and Socioeconomic Status: Two Oft Forgotten Factors in Child Metaphor Comprehension

**DOI:** 10.3390/children10121847

**Published:** 2023-11-25

**Authors:** Nausicaa Pouscoulous, Alexandra Perovic

**Affiliations:** Psychology and Language Sciences, University College London, Chandler House, 2 Wakefield Street, London WC1N 1PF, UK; n.pouscoulous@ucl.ac.uk

**Keywords:** metaphor, vocabulary, general language skills, socioeconomic status, gender, pragmatic development

## Abstract

Metaphor understanding can be tricky for children until mid-childhood, yet some research suggests that pre-schoolers are already competent. Many factors have been proposed to play a role in the development of metaphor comprehension. In this study we focus on two obvious contenders that have been overlooked in recent years: general language skills and socioeconomic status (SES). Two-hundred and seventy-two children, aged from 2;11 to 11;04 (146 girls) were recruited from 21 British schools and nurseries. Their SES was established using a composite measure linked to school location, while general language skills were assessed using a standardised measure of vocabulary comprehension. Novel metaphor comprehension was tested with a simple reference assignment task. Our study confirms that children interpret novel metaphors confidently from the age of 4. Our findings indicate that novel metaphor understanding is associated with age and, importantly, that it is linked to vocabulary skills, as well as SES, but not gender. These two factors should therefore be considered in future research on metaphor development, as well as intervention and education.

## 1. Introduction

Metaphor is prevalent in formal written language, as well as in the most mundane everyday speech. A child must therefore learn to understand metaphors to fully master linguistic communication. Traditionally, metaphor comprehension has been found to emerge relatively late—possibly not fully until mid-to-late childhood or even adolescence (for reviews see [1,2,3]). These early findings have been attributed in part to methodological issues, such as employing metalinguistic tasks, young children’s limited world knowledge and metaphorical expressions appearing with no, or little, context [4,5,6,7]. Indeed, recent findings show that even 3-to-5-year-old pre-schoolers understand metaphors in age-appropriate tasks that do not involve metalinguistic skills, such as explaining or paraphrasing the metaphors [6,7,8,9,10,11,12]. For instance, Pouscoulous and Tomasello [13] found that even young 3-year-olds were able to understand metaphors in a task where they had to pick a target toy referred to with a novel metaphorical expression. 

Apart from methodological limitations, findings on children’s metaphor understanding vary considerably depending on the type of metaphor investigated. For instance, the type of concept expressed metaphorically has a huge bearing on how early children are able to interpret them. Unsurprisingly, physical metaphors based on observable similarities are easier to grasp for younger children aged 4-to-7 than metaphors referring to emotional traits or abstract relations [14,15]. Additionally, pre-schoolers have not always acquired the world knowledge that would enable them to perceive the relevant abstract or psychological similarities between the topic and the vehicle [1,3,6]. More generally, the conceptual knowledge associated with both the topic and the vehicle of a metaphor plays a key part in reaching the non-literal interpretation of a metaphorical expression, as established by Gentner [16] with 4- and 7-year-olds, as well as adults. For instance, metaphor understanding has been shown to emerge contemporaneously within the same conceptual domain in 5-to-9-year-olds [8,17]. Furthermore, there are both theoretical and empirical reasons to expect that novel metaphors do not rely on the same interpretation process as conventional metaphors (or indeed idioms; e.g., [18,19,20,21]). While the meaning of novel metaphors is reconstructed online using the literal lexical meaning, world knowledge and relevant contextual information, that of conventional metaphors relies primarily on cultural knowledge—and for the child on whether and how much they have previously encountered the expression used in its figurative sense. Conventionality might therefore help processing in adults or older children who have had a lot of exposure to high frequency figurative meanings [22,23]. On the other hand, novel metaphors, which do not require prior exposure, may be more appropriate for studying children’s metaphorical abilities and establishing when they master the pragmatic inferential process enabling metaphor understanding [6].

Importantly, several psychological factors have also been put forward as playing a part in the emergence of metaphor understanding, such as the theory of mind and the ability to pass a standard false belief task ([24] but see [25,26] against this view). More recently, further skills have been claimed to be involved: for instance, analogical reasoning skills or the ability to attribute two labels to the same object, which improves drastically around the age of 4 [11,27], as well as executive control and the ability to inhibit the literal meaning of an expression to grasp its metaphorical interpretation, which also develops rapidly between 3 and 6 years of age [28,29]. 

Research on metaphor development in atypically developing children has also brought back into the spotlight the most basic, and possibly most crucial, influence: general linguistic abilities. Though the importance of language skills for typical metaphor comprehension throughout childhood was noted early on [1,3,4], this factor is primarily considered in the literature on atypical development. General linguistic skills, and in particular vocabulary and semantic knowledge, have now been argued to be the main factor accounting for poorer metaphor comprehension in children with autism [25,26,30], as well as children without autistic symptomatology, but experiencing linguistic difficulties [31,32,33,34,35]. It stands to reason that general language skills would have an impact on children’s metaphorical abilities. Yet, if this is the case, it should not be restricted to atypical language development. One would expect general linguistic competence to be linked to metaphor comprehension in typical language development, too. Indeed, a review by Matthews and colleagues [36] suggests that formal language is linked to pragmatic abilities in general. Yet, a more specific link between general language competence and metaphor understanding remains to be established in typical language development, both at younger and older ages.

The literature on language development also points to an important effect of socioeconomic status (SES) on vocabulary skills, particularly at younger ages, i.e., before the age of 3 [37]. For instance, compared to children from higher SES backgrounds, young children from lower SES and mid-SES backgrounds have been found to have slower rates of vocabulary growth [38,39], while their receptive vocabulary skills are affected by maternal education, ethnicity, and the frequency of home literacy activities [40]. Both the quantity [41] and the quality of language input [39,42] in households of different SES statuses have been argued to drive the differences in children’s language. These findings have been consistently replicated, including studies with large samples relying on a range of standardised language measures [43,44]. Few studies have directly investigated the relationship between SES and pragmatic skills. Law, McBean and Rush [45] report an exceptionally low performance on a standardised measure of pragmatics in school-age children growing up in socially disadvantaged areas. However, recent studies focusing on relevance implicatures found no influence of SES measured by parent education and income [46,47], though other factors, such as parents’ socio-cognitive engagement, have been reported to play a role [48]. It is important to note that the findings demonstrating the influence of SES on pragmatic language development (or lack thereof), and language and cognitive development in general, may well differ depending on the measures used. Interestingly, there are indications that metaphor interpretation may also be affected by SES, though research is sparse. A Canadian study whose focus was on bilingual metaphor comprehension reported that middle-class monolingual English-speaking 7-to-12-year-olds performed better than their working-class counterpart group [49]—although this finding was not replicated in other studies [46,50,51]. 

Yet another factor that may influence metaphor interpretation is gender. It is generally believed that girls show better language skills than boys, however, the results of studies involving different linguistic domains and participants of different ages show a complex picture [52]. Large-scale studies involving parent reports of language skills of young children aged 6 months to 2 years indicate a slight, but consistent advantage of girls over boys in the use of communicative gestures, expressive vocabulary and early word combinations [53,54,55]. Female advantage is still observed at later stages of language development—between the ages of 2 and 5—in different language domains, but this advantage disappears by the age of 6 [56]. Less is known about gender differences in pragmatic development, however. A recent surge of interest in potential gender differences in children with autism has seen an increase in studies comparing the mastery of figurative language in boys and girls. It suggests that autistic, as well as typically developing, girls might show some advantage over boys in understanding pragmatic phenomena such as metaphor and irony [57].

To investigate the role of general linguistic abilities and SES in metaphor comprehension, we recruited children from a range of SES backgrounds, and of a wider age range than in the literature reviewed above: aged 2;11 to 11;04. By including younger ages, we hope to target the population for whom differences in linguistic abilities and SES are likely to have a bigger impact. The children in our sample of 272—the largest in the literature to our knowledge—were assessed on a standardised measure of vocabulary comprehension, while their SES was established using the Index of Multiple Deprivation [58], a composite measure linked to school location, previously used in language acquisition research in the UK (see [44,59]). To assess metaphor comprehension we used the reference assignment paradigm designed by Pouscoulous and Tomasello [13], suitable even for the youngest participants, where the child is asked to hand the item corresponding to a metaphoric description out of two similar looking toys. Our aim was to isolate, as much as possible, the effect of vocabulary and SES; we therefore chose a design likely to minimise the influence of other confounding factors. We avoided metalinguistic tasks, kept the grammatical structure of the material very simple and used novel perceptual metaphors, which can be interpreted without previous exposure or learning, and were adapted to the world knowledge and linguistic abilities of young children from the age of 3. Additionally, the literal meaning of the target expressions was not involved in the metaphor comprehension task to avoid possible effects of inhibitory control, since it is known to be less developed in younger children [60,61]. Following Pouscoulous & Tomasello [13] and Bühler and colleagues [31], we included a naming-and-pointing picture book to check that children knew the vocabulary used in the task. When a child masters the literal meaning of an expression, we can assume that their performance on the metaphor task is a genuine indicator of their comprehension of a novel metaphor. If the child did not know the literal meaning of an expression it would prevent them from deriving the metaphorical interpretation, additionally, they could mistakenly overextend the conventional literal meaning of the expression in such a way that it includes the figurative one (early metaphor production is sometimes argued to result from such a process; for discussion, see [6]).

The current study thus aims to investigate whether, and how, general linguistic abilities, and more specifically vocabulary skills, as well as SES, influence children’s metaphor understanding. We expect to find a developmental trend, but also an effect of vocabulary proficiency and SES on metaphor comprehension. The effect of these two factors could be linked to each other: it would make sense for the effect of SES to be mediated through that of language abilities considering the correlation between the two often observed in the literature. Yet, these two factors may also influence metaphor understanding independently for different reasons. Additionally, we explore the possibility that there exist gender differences in children’s comprehension of metaphor, with girls performing better than boys on our novel metaphor task. 

## 2. Methods

### 2.1. Participants

Two hundred seventy-seven English-native speaking children, aged 35 to 136 months (M = 74.53; SD: 22.90) (2;11–11;04 years) were recruited from 14 primary state schools and 5 state and private nurseries in Southeast England (inner and greater London, and the counties surrounding greater London) and one primary school in Wales, in the United Kingdom. Efforts were made to recruit similar numbers of children in different age groups, however this was not always possible and they broke down as follows: 26 3-year-olds (2;11–3;11); 59 4-year-olds (4–4;11); 48 5-year-olds (5–5;11); 55 6-year-olds (6–6;11); 22 7-year-olds (7–7;11); 35 8-year-olds; 18 9-year-olds and nine 10–11-year-olds (10–11;3). Teachers were instructed to give consent forms to parents of children with no known developmental disorders, and with English as their only, or dominant, language (if exposed to more than one language at home). Two children were excluded following the first assessment after it was established that English was not their dominant language, and three more children were excluded for not having been administered the complete battery, leaving us with a sample of 272 children (146 girls). 

The study was approved by the University College London Research Ethics Committee. Prior to their taking part, verbal assent was obtained from all the children participating and written informed consent was obtained from their carers.

### 2.2. Materials and Procedure 

The assessments were administered by trained graduate students of speech and language therapy or language sciences. All the assessments were administered to all the children, in a fixed order: children were first presented with the standardised measure of vocabulary, followed by the metaphor comprehension task and finally the naming-and-pointing vocabulary book. Each participant was seen twice or three times for an individual session at their school/nursery. Some of the younger children were accompanied by a staff member who did not intervene during the task.

#### 2.2.1. Measures

##### Socioeconomic Background

To measure SES, we used the Index of Multiple Deprivation (IMD) [58], a measure of economic disadvantage that was previously used in language research in the UK [5,44]. IMD provides a set of relative measures of deprivation for different neighbourhoods, based on a range of domains, with different weighting: Income (22.5%); Employment (22.5%); Education, Skills and Training (13.5%); Health Deprivation and Disability (13.5%); Crime (9.3%); Barriers to Housing and Services (9.3%); and Living Environment (9.3%) [58]. The neighbourhoods are ranked and grouped into 10 deciles of deprivation, with 1 the most deprived (lower 10%), and 10 the least deprived (upper 10%). 

**Figure 1:**
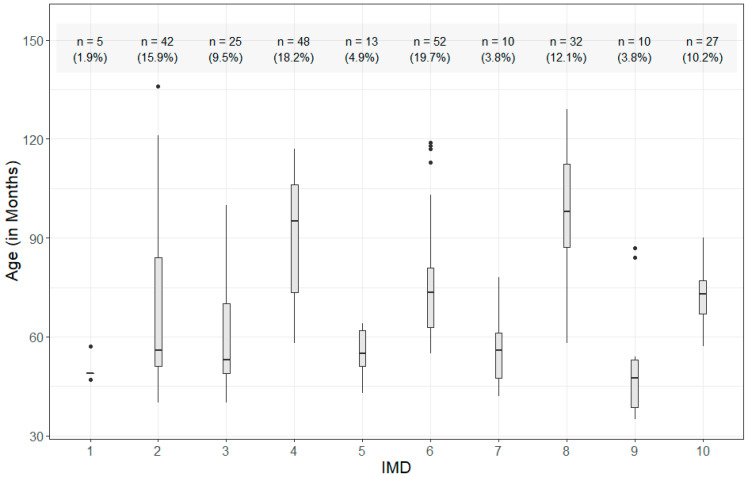
Mean age of participants in each IMD decile. Grey error bars represent 95% confidence intervals. Number of participants per IMD decile and percentage out of the total sample is presented at the top of the figure.

The IMD deciles were retrieved based on the schools’/nurseries’ postal codes and the Lower-Layer Super Output Areas (LSOA) code for England [62] and Wales [63]. The IMD deciles were relatively evenly spread in our sample (see Figure 1): 50% of the children were recruited from schools in the neighbourhoods with IMD deciles 1, 2, 3, 4 and 5, and 50% from schools in the neighbourhoods with deciles 6, 7, 8, 9 and 10. Only five children came from schools in the lowest first decile, but these were our youngest participants, aged 60 months and below. As observed in Figure 1, larger numbers of our youngest participants came from the neighbourhoods in the lower IMD deciles (e.g., 42 children from IMD decile 2 and 48 children from the IMD decile 4). This is in contrast to larger numbers of older participants having been recruited in the neighbourhoods in highest deciles (e.g., 32 children from IMD decile 8, and 27 children from the highest decile, IMD 10). This is observed in our correlation analyses, where IMD and Age were found to be weakly correlated (*r*(272) = 0.156; *p* = 0.010). No significant correlation was found between IMD and BPVS-2 (British Picture Vocabulary Scales 2) SS (*r*(272) = −0.028; *p* = 0.642), nor Age and BPVS-2 SS (*r*(272) = 0.082; *p* = 0.177). 

##### Vocabulary

Receptive vocabulary was assessed by the BPVS-2 [64], in which the child points to one of four pictures that matches the word uttered by the experimenter. This measure is commonly used with children as young as 3 years of age, and was administered to all our 272 participants.

Note that some children were also tested on a measure of receptive grammar (Test of Reception of Grammar 2 (TROG 2) [65]) and non-verbal reasoning (the Matrices subtest of the Kaufman Brief Intelligence Test (KBIT) [66]); however, since these measures are not standardised for children younger than 4, they were not included in the analyses.

##### Metaphor 

An adaptation of the Pouscoulous and Tomasello [13] design was used to assess metaphor comprehension. In this task, the experimenter asks the child to hand them one of two objects referred to by a metaphorical expression. Six pairs of similar looking toys were successively presented to each child. For each pair, the target item had the feature described by the metaphor, while the distractor object displayed another prominent, but irrelevant, characteristic. Following Pouscoulous and Tomasello, we used novel metaphors age-appropriate even for our younger participants; their target domains were body parts or clothing that young children are familiar with. For example, in one trial, participants were asked to hand the experimenter “the tower with the hat” when they were presented with two towers: one with a pointy roof and another one with a flat roof and a balcony (see Table 1 for the list of experimental items). The only cue to assign the correct referent was the metaphorical expression. The materials were validated on 10 adult English native speakers who showed a perfect performance. Translated equivalents were also previously used in a study by Pouscoulous and Tomasello [13], with German speaking adults (who performed perfectly) and 3-year-olds, as well as in a study by Bühler and colleagues [31], with 3-to-5-year-old Swiss German-speaking TD children and children with DLD. 

Metaphor trials were preceded by four familiarisation trials using literal expressions (e.g., “Give me the sheep with the belt/necklace”). During familiarisation the experimenter would point out mistakes; no corrections or comments were offered on the child’s choice during the metaphor test phase. No child made more than two errors on the familiarisation trial, so no participants were excluded from the analysis because they could not perform the task. The order of appearance of the metaphorical expression was randomised across the six test trials, while the target toy position was counterbalanced. 

We also created a picture book to assess children’s comprehension and production of the expressions used as targets in the metaphor task—both their literal and figurative meanings. It featured two sections (comprehension and production), each including one page per test metaphor in the metaphor task. Each page displayed images of an object representing what the metaphorical expression means literally (e.g., hat); what it referred to metaphorically (a roof); and a “nameless” object (e.g., a rare kitchen tool with no “child” label). The latter was included to prevent children from choosing the correct picture using deduction by elimination. Performance was correct for 90% or more of the words tested by the vocabulary picture book for children from all age groups, indicating that they mastered the literal meaning of the target expressions of the metaphor task. 

## 3. Results

Table 2 gives the results for the vocabulary measure, together with age of our sample, and Table 3 gives children’s performance on all different metaphors. 

Children’s performance was at ceiling on all metaphors with only a slightly lower performance on the metaphor for backpack (M = 0.87, SE 0.02). 

The data were analysed with the generalised linear mixed model procedure, GENLINMIXED, in the IBM SPSS statistical package version 27. The dependent variable was children’s performance on the metaphor task. Since this was a binary variable, i.e., participants’ answers were either correct or incorrect, the data were analysed using logistic regression with a logit link function in GENLINMIXED employing penalised quasi-likelihood estimation. Logistic regression is known to be better suited to binomially distributed data than standard ANOVAs [67]. Independent variables were Gender, entered as a categorical variable, and Age, BPVS score, and IMD score, which were continuous predictor variables, and thus were centred prior to being entered. With these variables entered into an initial model, participants and metaphor items were included as crossed random factors. However, this model failed to converge and after considering models with various combinations of random intercepts and random slopes for participants and items that were unsuccessful, we decided to treat metaphor items as a fixed effect due to the limited number of items included in this study (out of necessity, to keep the task manageable for very young children). Thus, the final model included participants as a random factor, metaphor items as repeated measures fixed factors and age, IMD, and BPVS as centred fixed factors. Treating metaphor items as a fixed effect will limit generalisation to other metaphors but does allow for the investigation of the influence of various factors on the comprehension of a sample of metaphors. In subsequent models, the inclusion of interactions between the fixed terms was not found to be justified as they did not account for the substantial explained variance. The model revealed significant effects of age F(1, 1622) = 32.097, *p* < 0.001; metaphor F(5, 1622) = 9.342, *p* < 0.001; BPVS-2 SS F(1, 1622) = 11.318, *p* < 0.001; and IMD F(1, 1622) = 8.392, *p* = 0.004. The effect of gender was not significant (F(1, 1622) = 1.024, *p* = 0.312. 

The effect of the metaphor is driven by participants’ ceiling performance on the M1 Tower, and the poorer performance of the M2 Backpack. Sidak-corrected post-hoc analyses confirmed that metaphor M1 Tower was comprehended significantly better than metaphor M2 Backpack (t(1622) = 5.340, *p* < 0.001), M4 Carrot (t(1622) = 3.737, *p* = 0.003) and M5 Carfoot (t(1622) = 3.713, *p* = 0.003). The metaphor M2 Backpack, on the other hand, was understood significantly worse than M3 Dog (t(1622) = 3.687, *p* = 0.003), M4 Carrot (t(1622) = 3.126, *p* = 0.018), M6 Bottle (t(1622) = 4.336, *p* < 0.001) and M1 Tower, as already observed. There were no other significant differences between the different metaphors tested.

To establish at which age the knowledge of metaphor becomes reliable, we examined the data of two youngest groups, aged 2;11–3;11 (n = 26) and 4;0–4;11 (n = 59). Amongst the youngest children, aged 2;11–3;11, just under half (42.3%) were classified as poor scorers—defined conservatively here as achieving only 3 or 4 correct out of the possible 6 (no child got less than 3 out of 6 correct). These poor scorers were equally spread across this age range. In the older group of 4;0–4;11-year olds, only 8 children were poor scorers (13.6%). Almost all of them were the youngest in this group, aged 53 months and younger. 

With regards to the effect of SES, out of the 27 children who were classified as poor scorers in our full sample of 272, only six children came from areas that can be defined as medium or higher SES (IMD deciles 5, 6, 7, 8, 9 and 10). The remaining 21 children, or 72% of our poor scorers, came from the lower SES areas (IMD deciles 1, 2, 3, 4). 

## 4. Discussion

In line with previous studies, our results indicate that children can understand novel metaphors from an early age. Metaphor comprehension on this reference assignment task is reliably predicted by age, vocabulary comprehension, and SES, but not gender. We discuss these in turn.

By the age of 4, our participants showed a confident performance on the task assessing novel metaphors. This confirms findings from other work using the same paradigm [13,31], as well as with other experimental designs [8,9,10,11,12]. While novel metaphors can be understood early, chronological age remains one of the strongest predictors of metaphor comprehension in the current study. This, too, resonates with the previous literature, which has consistently found that metaphor comprehension improves with age [11,68].

As expected, the other strongest predictor for children’s metaphor understanding was vocabulary comprehension. Our findings suggest that the language abilities that play a role in the children’s competence with metaphor comprehension are much more general than the specific vocabulary used in the experiment. Indeed, children answered more than 90% correctly in both comprehension and production in the pointing and naming vocabulary check, indicating that they mastered the literal vocabulary relevant to the task. The development of metaphor comprehension therefore appears to be linked to general language abilities and more specifically semantic skills. A review by Matthews and colleagues [36] already shows that competence in pragmatic measures is often associated with formal language in child language acquisition. Indeed, recent studies find such a link both for children’s implicature comprehension [69,70] and irony understanding [71,72,73]. Importantly, our study confirms that this point can also be made for metaphors specifically. 

Interestingly, although it is linked to general language skills, metaphor comprehension in this study is not driven by misunderstanding, difficulty with specific vocabulary or issues with the grammatical structures relevant to the task (and materials) at hand. While a link between vocabulary development and conventional metaphors stands to reason [22], accounting for it is less obvious for novel metaphors, which involve a pragmatic inference relying on literal meaning and context rather than exposure. Our findings therefore call for further investigation into how novel metaphor interpretation recruits general linguistic skills—over and above the specific expressions or structure used. 

The link between metaphor comprehension and general language abilities in typically developing children has interesting consequences for metaphor comprehension in atypically developing children, too. While, for instance, poor understanding of figurative language has traditionally been attributed to various aspects of autistic symptomatology (e.g., the theory of mind in [24]), a gradually stronger chorus of researchers suggests it might be mostly associated with the children’s poorer core language abilities [25,30,74] and particularly their semantic ability [26]. A link between metaphor understanding and general linguistic abilities has also been found in children with DLD [31], whose ability to understand another pragmatic phenomenon—implicatures—also depends on their general linguistic abilities [75]. Our findings therefore reinforce the need to look first at language ability when investigating poor metaphor comprehension—and perhaps pragmatic phenomena at large—in atypical development. In attaining full-fledged linguistic mastery, atypically developing children will be hindered by the same factors as typically developing children, above and beyond any other symptomatology.

Importantly, our findings also indicate a strong role of SES in metaphor comprehension development. The trend observed here is in line with Johnson [49]; however it contrasts with the results of some recent studies on metaphor comprehension development that did not find any effect of SES [50,51]. The SES of the children in these studies was probably too homogeneous to allow its influence on metaphor understanding to emerge (Valentina Bambini p.c.). Additionally, these studies tested 9–12-year-olds, and thus it is possible that the impact of SES has already diminished by this age or that it has been mitigated by the effect of schooling. The effect of SES on language development is most pronounced in early childhood [37]. It has also been reported that effective schooling may alleviate the impact of early disadvantage (e.g., see [76], for a large UK population study). We note that the few participants who performed at chance in our study came from the youngest age groups tested, and from the lowest SES backgrounds (IMD deciles 1, 2 and 3).

The lack of influence of SES on metaphor comprehension in some studies may also be related to the type of SES measures used. We used the IMD, a composite measure based on geographical location. In addition to standardly assessed income, education and employment status, it includes factors outside the home known to affect family wellbeing and impact child development. This measure may well prove to be a more reliable instrument for assessing SES than parental questionnaires.

A correlation between SES and vocabulary skills would not have been surprising considering the link between these two factors reported in the literature. Interestingly, we did not find a correlation. This suggests that the influence of SES is not—or not only—mediated by the effect of linguistic abilities, but impacts metaphor comprehension in children independently. Metaphor is a linguistic phenomenon, but establishing the link between the topic and the vehicle also requires world knowledge. Young children from affluent backgrounds may be more exposed to stimulating language-related and non-language-related activities in, and outside, their home, affecting their ability to comprehend novel metaphors. These activities are likely to impact the child’s world knowledge. Appropriate world knowledge seems key to understanding meaning shifts, if not most linguistic pragmatic inferences (see [77]). Understanding a novel metaphor, for instance, requires adequate knowledge of the topic and the vehicle domain (e.g., [6,7]). Importantly, a recent study brought to light the role of socio-cognitive engagement (e.g., joint parent-child interactions) for another type of pragmatic inference (relevance implicature) in 4-to-6-year-olds [48]. Although, the effect of socio-cognitive engagement was independent from SES in their study, it seems plausible it has (in part) driven the effect we observe here.

The lack of effect of gender on metaphor comprehension amongst our participants may seem surprising at first. While gender is argued to be a factor in early language acquisition, its effect appears to disappear by about 6 years of age [56]. Research focusing on gender differences in pragmatic skills is exceptionally sparse. Nonetheless, in a recent study where autistic and typically developing 9- to 11-year-olds were asked to paraphrase the true meaning of a figurative expression and identify the speaker’s intention, Sturrock and colleagues [57] found a slight advantage in girls over boys in both autistic and typically developing children. It is likely that the age range of our participants, and the nature of our task, facilitated the high performance of our participants masking any potential role of gender. While undoubtedly a fruitful area of research, the effect of gender in the acquisition of figurative language is only beginning to attract attention. We hope that more studies on pragmatic development will include this angle when reporting their findings, as insights from typical language development can also hugely benefit research on atypical language.

Further investigations could extend our findings. First, the undemanding nature of the task–motivated by our wish to include very young participants and eliminate various confounds while testing novel metaphors—resulted in reduced variance in our results: most children performed at ceiling. We believe that this, combined with a moderate number of trials per participant (6), may have influenced the magnitude of the effect, but not its direction [78]. Future research could therefore generalise our findings further by using different methods (e.g., picture selection tasks including a literal option such as [11]). Furthermore, if analogical reasoning plays a role in metaphor comprehension development [11], we could expect the children’s performance on more complex tasks to be related to measures of non-verbal abilities. Similarly, executive function might play an instrumental role in tasks that require inhibition of the literal meaning of the metaphorical expression [28,29].

This study focused on how general language abilities and SES are involved in metaphor comprehension in a large sample of young children from a range of diverse SES backgrounds. Overall, our results reveal that novel metaphor comprehension in nursery and school-age children is linked to age, vocabulary skills and SES. Of course, the importance of these factors does not preclude the possible involvement of other abilities in metaphor development (e.g., understanding pretence, double labelling, analogical skills), which deserve to be investigated carefully in future research. Future studies should aim to recruit very young participants (3-to-4-year-olds) from a wider range of SES areas. Finally, the importance of SES we uncover here leaves a lot of questions open about how precisely it influences children’s understanding of metaphor (and possibly other pragmatic phenomena). Nonetheless, our findings highlight how crucial it is to keep general language skills and SES in mind when investigating metaphors—as well as other pragmatic inferences—in typically and atypically developing children.

## Figures and Tables

**Table 1:** Metaphors with corresponding target and distractor toys adapted from Tomasello & Pouscoulous [13].

Metaphor	Target Toy	Distractor Toy
M1 The tower with the hat	Tower with a pointy roof	Tower with a balcony
M2 The car with the backpack	Car with a parcel on its roof	Car with a parcel inside
M3 The dog with the brown shoes	Dog with brown feet	Dog with a brown bow
M4 The carrot with the hair	Carrot with long fuzzy greens	Carrot circled by dark lines but with very short greens
M5 The car with the sick foot	Car with a missing wheel	Car with a missing door
M6 The bottle with the big belly	Round yellow bottle	White slender bottle

**Table 2:** Participants’ age and vocabulary comprehension scores. Notes: SD: standard deviation; SS: standard score. BPVS-2: British Picture Vocabulary Scales 2. The mean SS on BPVS-2 is 100, with SD = 15; range 40–160.

	n	Mean	SD	Range
Age in Months	272	74.53	22.90	35–136
BPVS-2 Raw score	272	70.24	23.43	20–140
BPVS-2 SS	272	107.38	11.87	73–150

**Table 3:** Mean proportion correct and standard error (SE) for each of the 5 metaphors.

Metaphor	Mean	SE
M1 Tower	0.99	0.01
M2 Backpack	0.87	0.02
M3 Dog	0.96	0.01
M4 Carrot	0.94	0.01
M5 Carfoot	0.92	0.02
M6 Bottle	0.97	0.01

## Data Availability

The participants of this study did not give written consent for their data to be shared publicly, so due to the sensitive nature of the research supporting data is not available.
